# Supplementary material for: Integrating contact tracing and whole-genome sequencing to track the elimination of dog-mediated rabies: An observational and genomic study
Source: eLife. 2023 May 25;12:e85262. doi: 10.7554/eLife.85262 (PMC10299823; doi:10.7554/eLife.85262)
Supplement: Supplementary file 2. — Exchange rate: 1 USD: 2296 Tsh (bank of Tanzania, 05/05/2022 https://www.bot.go.tz/). MoLDF = Ministry of Livestock Development and Fisheries, Tanzania; LTRA = Land transport regulatory authority; DoLD = Department of Livestock Development, Pemba. MSD = Medical Stores Department. LFO = Livestock Field Officer. *We do not include costs of vaccine collection from the airport. **each injection requires 5 minutes of health worker time and up to 8 injections per PEP course. [file elife-85262-supp2.docx]

**Table S2 Costs of rabies control and prevention activities.** Exchange rate: 1 USD: 2296 Tsh (bank of Tanzania, 05/05/2022 https://www.bot.go.tz/). MoLDF = Ministry of Livestock Development and Fisheries, Tanzania; LTRA = Land transport regulatory authority; DoLD = Department of Livestock Development, Pemba. MSD = Medical Stores Department. LFO = Livestock Field Officer. *We do not include costs of vaccine collection from the airport. **each injection requires 5 minutes of health worker time and up to 8 injections per PEP course.

| **Intervention** | **Cost variables** | **Unit Cost (USD)** | **Number** | **Source** |
| --- | --- | --- | --- | --- |
| Mass dog vaccination | Dog vaccine | 0.65 | Per dog | MoLDF |
|  | Consumables (syringes, needles) | 0.05 | Per dog | MSD price catalogue 2022/23 |
|  | Stationary (registers, certificates) | 4.36 | Per district | Local prices |
|  | Advertising for campaigns | 7.45 | Per vaccination day | DoLD |
|  | Transport (fuel) for team* | 7.45 | Per central point | DoLD |
|  | Assistant allowance | 2.18 | Per vaccination day | DoLD |
|  | LFO allowance | 13.07 | Per vaccination day | DoLD |
| Post-exposure vaccination | Consultation & wound care | 10.9 | Per patient | National health Insurance scheme |
|  | Post-exposure vaccine | 10.98 | Per vaccine vial | MSD price catalogue 2022/23 |
|  | Health worker time | 2.11** | Per patient | Tanzania Public service management and good governance |

### 
